# Assessing the quality of studies supporting genetic susceptibility and outcomes of ARDS

**DOI:** 10.3389/fgene.2014.00020

**Published:** 2014-02-06

**Authors:** Marialbert Acosta-Herrera, Maria Pino-Yanes, Lina Perez-Mendez, Jesús Villar, Carlos Flores

**Affiliations:** ^1^CIBER de Enfermedades Respiratorias, Instituto de Salud Carlos IIIMadrid, Spain; ^2^Research Unit, Hospital Universitario N.S. de CandelariaSanta Cruz de Tenerife, Spain; ^3^Research Unit, Hospital Universitario Dr. NegrinLas Palmas de Gran Canaria, Spain; ^4^Department of Medicine, University of CaliforniaSan Francisco, CA, USA; ^5^Keenan Research Center at the Li Ka Shing Knowledge Institute, St. Michael's HospitalToronto, ON, Canada; ^6^Applied Genomics Group (G2A), Genetics Laboratory, Instituto Universitario de Enfermedades Tropicales y Salud Pública de Canarias, Universidad de La LagunaSanta Cruz de Tenerife, Spain

**Keywords:** genetic susceptibility, acute respiratory distress syndrome, outcome, genetic factors, population stratification

## Abstract

The acute respiratory distress syndrome (ARDS) is a severe inflammatory disease manifested as a result of pulmonary and systemic responses to several insults. It is now well accepted that genetic variation influences these responses. However, little is known about the genes that are responsible for patient susceptibility and outcome of ARDS. Methodological flaws are still abundant among genetic association studies with ARDS and here, we aimed to highlight the quality criteria where the standards have not been reached, to expose the associated genes to facilitate replication attempts, and to provide quick-reference guidance for future studies. We conducted a PubMed search from January 2008 to September 2012 for original articles. Studies were considered if a statistically significant association was declared with either susceptibility or outcomes of all-cause ARDS. Fourteen criteria were used for evaluation and results were compared to those from a previous quality assessment report. Significant improvements affecting study design and statistical analysis were detected. However, major issues such as adjustments for the underlying population stratification and replication studies remain poorly addressed.

## Introduction

Acute lung injury (ALI) and its severe form, the acute respiratory distress syndrome (ARDS), are characterized by acute diffuse lung inflammation and non-cardiogenic pulmonary edema resulting from increased capillary-alveolar permeability. While ALI and ARDS terms continue to be used in the medical literature, their definition criteria were recently revised, although a consensus has not been reached (Ranieri et al., 2012; Villar et al., 2013). New definitions support the categorization of ARDS based on the hypoxemia severity under mechanical ventilation, as well as on other physiological and clinical parameters, discouraging the use of ALI as one of the categories. Hereafter, we will refer to this constellation of syndromes using the term ARDS, irrespective of the classification used by the studies reviewed (Bernard et al., 1994). ARDS shows profound incidence variability across countries (Rubenfeld et al., 2005; Villar et al., 2013), and it is unknown whether differences also exist among ethnic groups (Martin et al., 2003; Erickson et al., 2009; Linko et al., 2009; Villar et al., 2011) and the extent to which demographic, cultural, economical, and health system particularities might underlie such differences.

Predisposing genetic factors can interact with the environment to determine the diversity of clinical manifestations, the response to treatment and outcomes among ARDS patients (Cobb and O'Keefe, 2004; Villar et al., 2004; Rahim et al., 2008). Exposing those genetic factors might reveal therapeutic targets and a foundation to predict ARDS susceptibility and outcomes. Association studies have been widely used for detecting common, low-penetrant, genetic variants that are suggested to contribute to the genetic architecture of complex diseases (Khoury and Yang, 1998), including ARDS (Flores et al., 2008). For ARDS, these studies have mostly focused on particular biological candidates and, only recently, have explored the entire genome (Christie et al., 2012). We have previously assessed the quality of statistically significant associations of genetic variants with ARDS from 1996 to 2008 based on major recommendations that support study robustness (Flores et al., 2008). We hypothesized that, despite this previous evaluation and the availability of well-known standard guidelines (Janssens et al., 2011), many association studies in this field continue to be performed without awareness of minimal standards and that methodological flaws are still abundant. Here, we aimed to identify those quality criteria where the standards have not been reached, to expose the associated candidate genes to facilitate replication studies, and to create a guidance framework for ongoing and future studies. For that, we have critically assessed statistically significant candidate-gene associations with susceptibility or outcome of all-cause ARDS from 2008 to 2012 using 14 major quality control criteria, and compared the updated results with our previous evaluation (Flores et al., 2008).

## Materials and methods

### Literature search

We have previously assessed the quality of genetic association studies supporting susceptibility and/or outcome in adult ARDS patients of the period of 1996–2008 (Flores et al., 2008). We have now conducted a PubMed search from January 2008 to September 2012 by utilizing the same keyword combinations for querying (“polymorphism” and “acute lung injury,” “polymorphism” and “ARDS,” and “polymorphism” and “acute respiratory distress syndrome”). Because of the plausibility that a fraction of risk variants for ARDS susceptibility could be also risk factors for outcomes, both possibilities were jointly analyzed. The retrieved references were then manually reviewed. Excluding meta-analysis, those reporting statistically significant associations in adults (*p* ≤ 0.05) for any cause of ALI or ARDS irrespective of the type of genetic variants associated, and published in English, were reviewed by three of the authors. We are aware that a number of such reported associations might be false positives. However, this threshold for significance is preferable over a more conservative strategy at this stage of field development (Thomas and Clayton, 2004). Finally, we considered the gene as the unit of replication (Neale and Sham, 2004).

### Study assessment

For simplicity, we focused on the 14 most relevant criteria, previously utilized by us in Flores et al. (2008), modifying the exhaustive list provided by Chanock et al. (2007), scoring each item as present or absent. Chi-squared tests were performed in SPSS (SPSS Inc., Chicago, IL).

### Gene coverage in genotyping arrays

Gene coverage was calculated with the tagger tool (Barrett et al., 2005) for SNPs with minor allele frequency >5% in the gene region captured directly and indirectly by the genome-wide genotyping array utilized (with a multi-marker *r*^2^ ≥ 0.8).

## Results

The PubMed search on the period 2008–2012 allowed a closer review of 27 original articles reporting statistically significant association findings on 31 candidate genes with susceptibility and/or outcomes of all-cause ARDS (Table S1), and the first genome wide association study (GWAS) for this syndrome (Christie et al., 2012). The latter was excluded from the evaluation as its quality control assessment differs substantially from those applied to candidate-gene studies. A complementary search querying for the syndrome name in the HuGeNet Navigator (Yu et al., 2008) gave overlapping results, showing studies for additional genes albeit all reporting statistically non-significant findings. We, therefore, continued the quality assessment based on the PubMed search.

Seventeen studies (63%) provided statistically significant findings with a case-control design and ten (37%) with a cohort. These were based on a median sample size of 251 cases [interquartile range (*P*_25_–*P*_75_): 84–365] and 288 controls (*P*_25_–*P*_75_: 190–724) in case-control studies, whereas for cohort studies the median sample size was 145 patients (*P*_25_–*P*_75_: 118–215). In this period, almost all studies (96%) appropriately described demographical and clinical data for cases and all had an adequate characterization of the control group (47.1% of them utilized healthy subjects or population-based controls and 52.9% opted to use at risk patients as controls). However, only 50% of the studies explored their power to detect statistically significant findings.

While roughly a third of studies (35%) focused on a single variant of the gene under study, the majority (65%) analyzed several polymorphisms attaining appropriate gene coverage of common variation by means of linkage disequilibrium (LD)-based methods. In most cases (74%), the studies allowed to unambiguously identify the genomic location of the associated variant(s) on public resources. Similarly, most studies declared that Hardy-Weinberg equilibrium expectations were assessed (93%), and that further genotyping error checks were implemented during the study (59%). Almost half of the studies (48%) stated that genotyping was performed blind to the disease status of samples.

**Figure 1:**
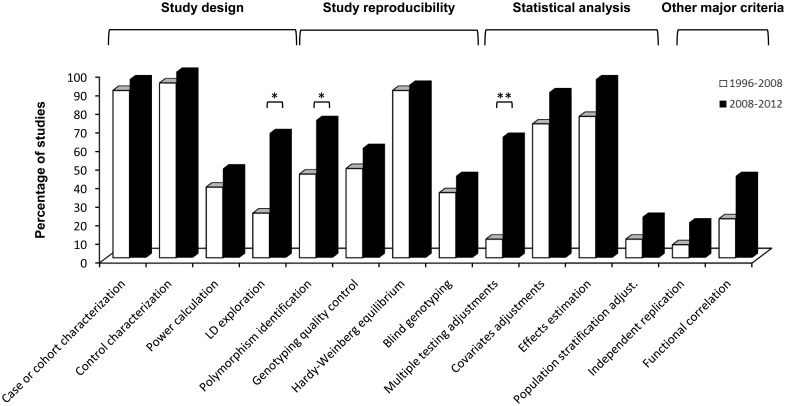
**Histogram comparing quality control scores of association studies in ARDS published from 1996 to 2008 (taken from Flores et al., 2008) and from 2008 until present.** Statistically significant improvements affected criteria relevant to study design (LD exploration), study reproducibility (polymorphism identification) and statistical analysis (multiple testing adjustments). ^*^p-value ≤ 0.05; ^**^p-value ≤ 0.001.

Focusing on the statistical analyses, 65% of the studies that needed to control type-I error due to multiple hypothesis testing did so, and 89% included covariates in the regression analyses. The magnitude of effects was appropriately reported in terms of hazard ratios (HRs) or odds ratios (ORs) in almost all reviewed studies (96%) (Table S1). The adjustment for population stratification and replication, in at least an independent study sample, were declared only in 22 and 19% of the studies, respectively, two major issues that has not improved over the years (Flores et al., 2008) (Figure 1). Similarly, almost half of the studies (44%) pursued the functional significance of associated variants.

On a side-by-side comparison of the two periods reviewed to date (i.e., 1996–2008 reviewed by Flores et al., 2008 and this one from 2008 to 2012), significant improvements in the quality of the published studies were observed in the most recent period (Figure 1) affecting study design, study reproducibility, and statistical analysis. These improvements were due to an increase of studies exploiting the available tools for LD exploration to efficiently select the genetic variants (from 24 to 67%, chi-squared *p* = 0.003); controlling type-I error by incorporating multiple testing adjustments on the analyses (from 10 to 65%, chi-squared *p* = 0.0003); and accurately identifying the genomic location of the associated variant(s) (from 45 to 74%, chi-squared *p* = 0.033).

## Discussion

**Figure 2:**
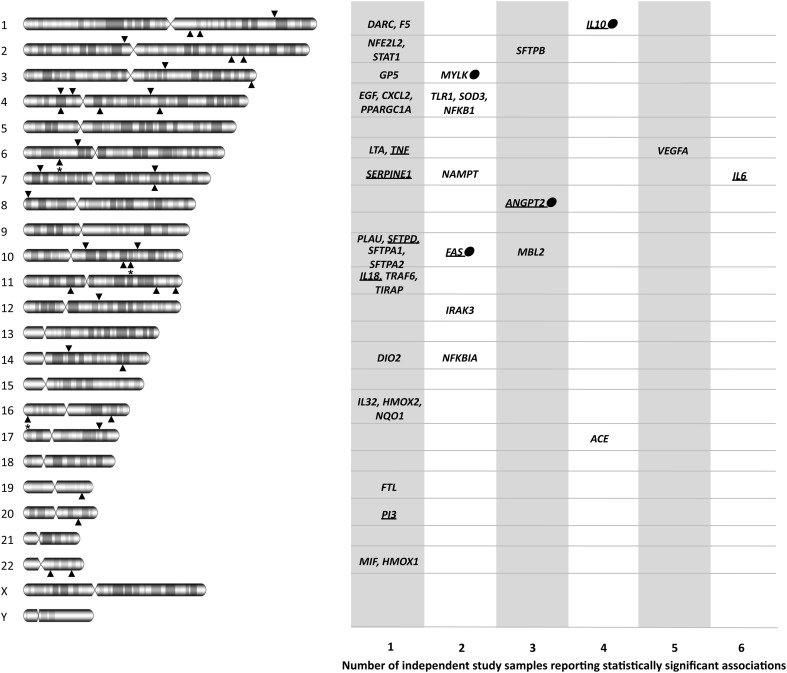
**Diagram showing the official gene symbols for the 41 candidate genes associated with ARDS susceptibility and outcomes, depicting both chromosome locations and the number of study samples with statistically significant associations.** For each chromosome, lower arrowheads indicate the location of genes with a single sample association, and upper arrowheads indicate the location of genes with statistically significant association findings in at least two study samples. Arrowheads with asterisk indicate more than one gene in that region. Dots denote that the gene was replicated in the only GWAS of ARDS published to date. Underlined gene names indicate that the product has been suggested as a biomarker for ARDS or its progression in at least one study. *ACE*, angiotensin I converting enzyme; *ANGPT2*, angiopoietin 2; *CXCL2*, chemokine (C-X-C motif) ligand 2; *DARC*, duffy blood group, chemokine receptor; *DIO2*, deiodinase, iodothyronine, type II; *EGF*, epidermal growth factor; *F5*, coagulation factor V (proaccelerin, labile factor); *FAS*, TNF receptor superfamily, member 6; *FTL*, ferritin, light polypeptide; *GP5*, glycoprotein V (platelet); *HMOX1*, heme oxygenase 1; *HMOX2*, heme oxygenase 2; *IL6*, interleukin 6; *IL10*, interleukin 10; *IL18*, interleukin 18; *IL32*, interleukin 32; *IRAK3*, interleukin-1 receptor-associated kinase 3; *LTA*, lymphotoxin alpha; *MBL2*, mannose-binding lectin 2; *MIF*, macrophage migration inhibitory factor; *MYLK*, myosin light chain kinase; *NAMPT*, nicotinamide phosphoribosyltransferase; *NFE2L2*, nuclear factor (erythroid-derived 2)-like 2; *NFKB1*, nuclear factor of kappa light polypeptide gene enhancer in B-cells 1; *NFKBIA*, nuclear factor of kappa light polypeptide gene enhancer in B-cells inhibitor alpha; *NQO1*, NAD(P)H dehydrogenase, quinone 1; *PI3*, peptidase inhibitor 3; *PLAU*, plasminogen activator, urokinase; *PPARGC1A*, peroxisome proliferator-activated receptor gamma, coactivator 1 alpha; *SFTPA1*, surfactant protein A1; *SERPINE1*, serpin peptidase inhibitor, clade E (nexin, plasminogen activator inhibitor type 1), member 1; *SFTPA2*, surfactant protein A2; *SFTPB*, surfactant protein B; *SFTPD*, surfactant protein D; *SOD3*, superoxide dismutase 3; *STAT1*, signal transducer and activator of transcription 1, 91kDa; *TIRAP*, toll-interleukin 1 receptor (TIR) domain containing adaptor protein; *TLR1*, toll-like receptor 1; *TNF*, tumor necrosis factor; *TRAF6*, TNF receptor-associated factor 6; *VEGFA*, vascular endothelial growth factor A.

We have assessed the evidence obtained during 2008–2012 from ARDS candidate-gene association studies and compared them with our previous assessment to objectively evaluate the evolution of the field, especially in light of the methodology applied in genetic susceptibility studies. In total, including the evidence accumulated before 2008 (Flores et al., 2008), 56 studies on 41 candidate genes reported statistically significant associations with susceptibility or outcomes of all-cause ARDS (Figure 2).

We detected significant improvements affecting the exploitation of resources for LD exploration, the inclusion of multiple testing adjustments, and the way studies identified the associated variants by established recommendations. This was also extensible to sample sizes for case-control designs, as these have roughly doubled their median sample by group compared to studies published before 2008. Despite this improvement, replications in independent studies are needed to improve the association reliability. Worth noting, the diversity of samples has increased over the years, so that across all published studies a few have focused on African-Americans (6.6%), while the majority continues to use Europeans (66.7%), East Asians (15%), or multiethnic samples (11.7%). While all these improvements are stimulating, a downside continues to be recognized on the adjustment for population stratification and replication attempts, as these were conducted in less than a fifth of all reviewed reports.

The identification of genuine gene associations with ARDS relies on conducting more replication studies, albeit without sacrificing study robustness, as only a few associated genes have been replicated to date (Figure 2). Among those genes, *ACE* was associated several times and a meta-analysis was recently published (Matsuda et al., 2012). Although results should be taken with caution because of power limitations, they revealed variable effects of an *ACE* polymorphism with ARDS mortality, present in East Asians but lacking in Europeans. This illustrates the growing evidence supporting that genetic risks may be population-specific, either because of gene-gene or gene-environment interactions or because of frequency effects (Need and Goldstein, 2009). Given that we are far from having a complete list of ARDS genes, and that an incomplete overlap of genetic risks between populations is expected, the study of samples of diverse ancestry should be encouraged in future studies. It must be noted that across all reviewed studies, genetic associations with ARDS susceptibility or outcomes with opposite effects in different ancestry groups were absent, despite differences by the ARDS triggering insult have been detected (Christie et al., 2008). One major issue that is determinant of the robustness of association studies with unrelated individuals is the assessment and adjustment of results for the underlying (sometimes cryptic) population stratification, which is usually based on data from independent genetic polymorphisms (Price et al., 2006). Still today, more than 80% of the published association studies in ARDS did not apply such an approach, despite few dozen of very informative genetic variants (termed AIMs) have demonstrated their utility in specific populations (Pino-Yanes et al., 2011; Galanter et al., 2012). As the studies that focus on particular genomic regions will continue to be relevant in the field (Chanock et al., 2007), population stratification effects should be minimized in future association studies, irrespective of the study population being assessed. Therefore, it becomes essential to develop efficient and straightforward methods that: (1) could be applied to different populations and be universally used, and (2) could assist researchers to easily select a reduced set of AIMs to accurately assess ancestry maintaining affordable costs. Such tools would be useful to validate study robustness as well as to address the biological differences between populations, and whether these may trigger disparities in ARDS susceptibility or outcomes. It must be noted; however, that population stratification also introduces non-genetic effects that will not be addressed by these methods. It is expected that analyses of these effects and interactions will bring new opportunities and challenges in the field (Rotimi and Jorde, 2010).

Establishing the association of genes with ARDS susceptibility is only the beginning of a process that should continue with the discovery of the causal genetic variants. The challenge continues to be the validation of existing and novel ARDS associations via robust studies, and future and ongoing studies should amend the critical issues here recognized. In this effort, new technologies are allowing a faster field development by means of genome-wide studies, either using genotyping arrays or exome/whole genome sequencing. GWAS are as efficient as candidate-gene studies for detecting weak effect risks, not requiring a previous hypothesis of the biological processes related to the trait. They have allowed to identify new disease genes never anticipated and led to new hypothesis and perspectives about disease pathogenesis (Marchini and Howie, 2010). Despite that, GWAS have major limitations including high costs, usually impacting on the sample size, the statistical burden and the gene coverage. In addition, most commercial platforms may offer less coverage for the gene(s) of interest compared to that achieved in optimal candidate-gene studies, which can substantially impact study power (Voight et al., 2012). The first GWAS of ARDS was recently published by Christie et al. (2012), revealing *PPFIA1* as a novel susceptibility gene involved in cell adhesion and cell-matrix interactions, and suggesting many others with putative functional roles. This study also replicated the association of four candidate genes including *IL10*, *MYLK*, *ANGPT2*, and *FAS*. This may suggest that all other candidate gene associations should be considered false discoveries. However, one explanation for this inconsistency could be also the insufficient GWAS coverage of the non-associated candidate genes (average ≈57%; Table S2). Whatever the case, commercial platforms will only allow studying a fraction of the millions of existing genetic variants (Abecasis et al., 2012), and it is anticipated that the associations to be revealed will only explain a small component of the disease (Manolio et al., 2009). Only complete re-sequencing of individual genomes will guarantee the analysis of all genetic variation.

Here we have shown that the field still faces several methodological challenges, and in the clinical arena there are key issues to be improved in order to fully understand the genetic processes underlying ARDS. Misclassification of phenotypes can lead to significant reduction in statistical power to detect true genetic associations, therefore it becomes necessary a better and more homogeneous patient classification. This could be achieved by combining the clinical information with different integrative approaches, those based on the determination of the causal microorganisms by means of metagenomics (Lysholm et al., 2012) or performing gene expression profiling among patients (Hu et al., 2012), to name a few. As a proof of concept, in a recent study by O'Mahony et al. (2012), only when the samples were restricted to the more severe phenotype, new associations were revealed and previous findings were replicated. Furthermore, quantitative phenotypes could be utilized for association testing, such as ventilator-free days (Kangelaris et al., 2012) or ideally other traits that are closer to the genotype. This possibility has been explored in the field with striking (Wurfel et al., 2008) and replicable results (Pino-Yanes et al., 2010). Additionally, the selection of the control samples remains a challenge; it is not an easy task and not a single design is free of bias. The use of either healthy subjects or at-risk individuals is common among the reviewed studies. An alternative solution can be the utilization of both types of controls to reduce selection biases and be able to confidentially assess the quality of the genotypic data. This strategy has been used (Song et al., 2010), and will surely reduce the chances that risk variants reported are causally associated with a confounder and not with ARDS.

In summary, the methodology for assessing genetic risks in complex diseases is under development. For ARDS, we conclude that the main challenge continues to be in providing an analytically rigorous methodology (adjusting for population stratification, relatedness, and technical quality) accompanied by independent replication and mechanistic explanations for the results provided. Still today, the evidence supporting the genetic associations with ARDS susceptibility or outcomes is at best uncertain, given the limited statistical power of most studies and the effects expected for genetic variants involved in complex traits. To guarantee proper and high quality studies on genetic susceptibility and outcomes, we strongly encourage the use of large and well-defined collection of samples. Consequently, a shift toward the establishment of international consortia will be necessary.

## Author contributions

All authors contributed equally in the assessment design and read and approved the final manuscript.

## Conflict of interest statement

The authors declare that the research was conducted in the absence of any commercial or financial relationships that could be construed as a potential conflict of interest.
