# Supplementary material for: Assessing the quality of studies supporting genetic susceptibility and outcomes of ARDS
Source: Front Genet. 2014 Feb 6;5:20. doi: 10.3389/fgene.2014.00020 (PMC3915143; doi:10.3389/fgene.2014.00020)
Supplement: Supplementary file 1 [file DataSheet1.PDF]

Supplementary Material

Assessing the quality of studies supporting genetic susceptibility and outcomes of ARDS

Marialbert Acosta-Herrera,[1,2,3] Maria Pino-Yanes,[1,2,4] Lina Perez-Mendez,[1,2] Jesús Villar,[1,3,5] Carlos Flores*[1,2,6]

(1) CIBER de Enfermedades Respiratorias, Instituto de Salud Carlos III, Madrid, Spain;
(2) Research Unit, Hospital Universitario N.S. de Candelaria, Santa Cruz de Tenerife, Spain;
(3) Research Unit, Hospital Universitario Dr. Negrin, Las Palmas de Gran Canaria, Spain;
(4) Department of Medicine, University of California,San Francisco, CA, USA;
(5) Keenan Research Center at the Li Ka Shing Knowledge Institute, St. Michael´s Hospital, Toronto, Canada.
(6) Applied Genomics Group (G2A), Genetics Laboratory, Instituto Universitario de Enfermedades Tropicales y Salud Pública de Canarias, Universidad de La Laguna, Santa Cruz de Tenerife, Spain.

***Correspondence:** Carlos Flores, PhD, Unidad de Investigación, Hospital Universitario N.S. de Candelaria, Carretera del Rosario s/n, 38010, Santa Cruz de Tenerife, Spain. Phone: (+34) 922-602938. Fax: (+34) 922-600562. e-mail: cflores@ull.edu.es

1. Supplementary Tables

1.1. Supplementary Table S1

Table S1. Studies reporting positive associations with susceptibility and/or outcomes of all-cause Acute Respiratory Distress Syndrome in the period 2008-2012 and the details for the variants associated (top significance reported and its effect).

Year	Gene(s)	Associated variant(s)	Top p-value	Effect (95% CI)[a]	Sample size (case:control)[b]	Sample size (cohort)[b]	Trait(s) positively associated[c]	Population[b]	Ref.
2008	*FTL*	rs905238	9.0E-03	2.44 (1.29-4.63)	104:193		ARDS	E	(Lagan,2008)
2008	*HMOX2*	rs1051308	4.0E-02	0.36 (0.17-0.80)	104:193		ARDS	E	(Lagan,2008)
2008	*TLR1*	rs5743551	4.1E-02	3.40 (1.59-7.27)	138:167		ARDS	E	(Wurfel,2008)
2009	*ANGPT2*	rs2515475	4.0E-03	1.79 (1.21-2.65)	449:1080		ARDS	E	(Su,2009)
		rs2959811	4.0E-02	1.35 (1.02-1.81)					
		rs2515475-rs2959811	4.0E-03	1.90 (1.23-2.92)					
2009	*EGF*	rs4444903	5.0E-03	1.64 (1.17-2.31)	416:1052		ARDS, extubation rate, VFD	E	(Sheu,2009)
		rs2298991	1.9E-02	1.50 (1.07-2.11)					
		rs7692976	5.0E-03	1.64 (1.17-2.31)					
		rs4698803	2.5E-02	0.67 (0.48-0.96)					
		rs4444903-rs2298991-rs11568893-rs7692976-rs469880-rs65334853	2.2E-02	0.64 (0.44-0.94)					
2009	*HMOX1*	rs3074372	4.0E-03	0.66 (0.47-0.88)	437:1014		ARDS	E	(Sheu,2009)
2009	*LTA*	rs1041981	3.0E-03	16.50 (2.40-112.80)		34	Duration of mechanical ventilation, ICU stay, mortality	A	(Liu,2009)
2009	*NQO1*	rs689455	2.9E-02	0.49 (0.25-0.93)		133 (AA), 124 (E)	ARDS	E, AA	(Reddy,2009)
2009	*PI3*	rs1983649	3.4E-02	1.37 (1.02-1.84)	449:1031		ARDS	E	(Tejera,2009)
		rs2664581	4.0E-03	1.65 (1.16-2.34)					
2009	*SERPINE1*	rs1799768	9.0E-03	9.95 (1.79-55.28)		52	VFD, OFFD, mortality	E	(Tsangaris,2009)
2009	*SOD3*	rs2536512-rs8192291-rs2695232-rs2855262	1.0E-02	NA	252:179, 157:179		ARDS, VFD, mortality	E	(Arcaroli,2009)
2010	*FAS*	rs17447140	4.0E-03	NA	324:294, 194:463		ARDS	E	(Glavan,2010)
		rs2147420	4.0E-02	NA					
		rs2234978	2.0E-02	NA					
		rs1051070	4.0E-02	NA					
		rs2147420-rs2234978-rs1051070	7.0E-03	1.73 (1.16-2.57)					
2010	*IL18*	rs1946518	6.0E-04	0.42 (0.26-0.70)		198	ARDS	A	(Chen,2010)
2010	*TIRAP*	rs595209	4.1E-03	1.43 (1.12-1.82)	278:288[d]		ARDS	A	(Song,2010)
		rs8177375	9.8E-04	1.81 (1.30-2.69)					
2010	*TLR1*	rs5743551	3.0E-03	2.68 (1.40-5.12)		218	ARDS	E	(Pino-Yanes,2010)
2011	*ACE*	rs4646994	8.0E-03	8.80 (1.75-44.07)		101	Mortality	A	(Lu,2011)
2011	*ANGPT2*	rs2442598	2.5E-05	2.73 (1.71-4.35)	61:161 (AA), 600:2266 (E)		ARDS	E, AA	(Meyer,2011)
		rs1868554	3.3E-05	2.60 (1.66-4.09)					
2011	*DIO2*	rs225015	3.0E-03	NA	41:187 (AA), 78:188 (E)		ARDS	E, AA	(Ma,2011)
		rs225014	4.0E-03	0.53 (0.34-0.82)					
		rs225013	1.0E-03	NA					

		rs225010	2.0E-03	NA					
		rs225014-rs12885300	5.0E-03	NA					
2011	*GP5*	rs2185479	6.0E-05	6.17 (2.54-15.00)	61:161 (AA), 600:2266 (E)		ARDS	E, AA	(Meyer,2011)
2011	*IL6*	rs1800795	2.0E-03	0.25 (0.07-0.79)		306	ARDS	E	(Martin-Loeches,2011)
2011	*IL10*	rs1800896	2.0E-03	0.68 (0.53-0.87)	314:210		ARDS, mortality	A	(Jin,2011)
2011	*IL32*	rs12934561xrs1554999	3.2E-03	0.40 (0.30-0.80)	251:258		ARDS	E	(Arcaroli,2011)
		rs2239303-rs12934561	8.0E-03	NA					
2011	*IRAK3*	rs10506481	2.1E-02	2.50 [1.15-5.47]		214	ARDS	E	(Pino-Yanes,2011)
		rs1732886	3.3E-02	2.57 [1.02-6.10]					
		rs1732888	9.0E-03	NA					
2011	*NFKB1*	rs28362491	3.0E-02	9.43 [1.21-73.20]	379:793		ARDS	E	(Bajwa,2011)
2011	*PPARGC1A*	rs6892794	7.8E-05	2.45 [1.57-3.83]	61:161 [AA], 600:2266 [E]		ARDS	E, AA	(Meyer,2011)
2011	*SFTPD*	rs721917	3.2E-02	1.92 [1.06-3.48]	26:656		ARDS	E	(Garcia-Laorden,2011)
2011	*SFTPA1*	rs1059047-rs1136450-rs4253527	4.0E-03	3.89 [1.56-9.72]	26:656		ARDS	E	(Garcia-Laorden,2011)
	SFTPA1-SFTPA2	rs1059047-rs1136450-rs4253527-rs1059046-rs17886395-rs1965708	6.0E-04	5.83 [2.12-16.04]					
2011	*STAT1*	rs6734110	4.9E-05	7.29 [2.79-19.00]	61:161 [AA], 600:2266 [E]		ARDS	E, AA	(Meyer,2011)
2011	*VEGFA*	rs3025039	4.0E-02	3.85 [1.05-14.06]	170:170		ARDS	A	(Yang,2011)
2012	*DARC*	rs2814778	2.0E-02	0.74 [0.58-0.96]		132	VFD, OFFD, mortality	AA	(Kangelaris,2012)
2012	*TRAF6*	rs4755453	1.2E-03	0.71 [0.58-0.82]	272:276		ARDS	A	(Song,2012)
2012	*ANGPT2*	12-SNP ht[e]	1.0E-06	NA	61:161 [AA], 74:150 [E]		ARDS	E, AA	(Meyer,2012)
2012	*IL6*	rs12700386-rs3087221-rs2069824	3.8E-03	NA	61:161 [AA], 74:150 [E]		ARDS	E, AA	(Meyer,2012)
2012	*IL10*	7-SNP ht[f]	2.1E-02	NA	61:161 [AA], 74:150 [E]		ARDS	E, AA	(Meyer,2012)
2012	*IRAK3*	15-SNP ht[g]	4.4E-02	NA	61:161 [AA], 74:150 [E]		ARDS	E, AA	(Meyer,2012)
2012	*VEGFA*	rs3025030-rs3025035-rs3025039	8.8E-03	NA	61:161 [AA], 74:150 [E]		ARDS	E, AA	(Meyer,2012)
2012	*NFKBIA*	rs3138056-rs3138055-rs696-rs1022714	4.2E-02	NA	61:161 [AA], 74:150 [E]		ARDS	E, AA	(Meyer,2012)

[a]Odds or hazard ratio, as appropriate, and its 95% confidence interval.
[b]AA: African-Americans; E: Europeans; A: East Asians.
[c]ARDS: Acute respiratory distress syndrome; ICU: Intensive care unit; VFD: Ventilator free days; OFFD: organ failure free days.
[d]298 Healthy controls were also analyzed.
[e]haplotypeinvolved the following SNPs: rs2922889-rs2515464-rs6990020-rs2515466-rs2442608-rs1868554-rs10503371-rs12674822-rs1984857-rs17077419-rs2959813-rs2442598
[f]haplotype involved SNPs: rs1554286-rs3024490-rs3024489-rs1800872-rs1800871-rs1800896-rs1800893
[g]haplotype involved the following SNPs: rs1168774-rs17767298-rs12314460-rs1185630-rs1882200-rs1617632-rs1152888-rs1168643-rs1370128-rs3782347-rs17826057-rs11176095-rs1152912-rs1152913-rs1152918
Notes: More than one study sample size indicates the replication of the association in an independent sample; SNPs associated as part of haplotypes are separated by dashes; SNPs associated as part of SNP-SNP interactions are separated by 'x'; NA: data not available, or not indicated in the original article.

1.2. Supplementary Table S2

Table S2. Coverage of common variants for Europeans by the Illumina HumanQuad610 BeadChip for all candidate genes positively associated to date with Acute Respiratory Distress Syndrome susceptibility or outcomes.

Gene	Chromosome	Chr. position[a]	Common SNPs[b]	#Array SNPs	#taggedSNPs[c]	Coverage (%)[d]
DARC	1	159173803-159176290	22	2	2	9
F5	1	169481192-169555769	309	39	285	92
IL10	1	206940948-206945839	21	9	21	100
NFE2L2	2	178095031-178129859	40	5	17	43
SFTPB	2	85884440-85895864	17	1	1	6
STAT1	2	191833762-191878976	86	14	61	71
MYLK	3	123331143-123603149	311	46	198	64
GP5	3	194115550-194119995	6	1	1	17
PPARGC1A	4	23793644-23891700	208	31	128	62
SOD3	4	24797085-24802467	15	1	1	7
TLR1	4	38797876-38806412	31	3	28	90
CXCL2	4	74962752-74964997	2	2	2	100
NFKB1	4	103422486-103538459	186	18	162	87
EGF	4	110834040-110934118	168	23	123	73
LTA	6	31539876-31542101	20	9	7	35
TNF	6	31543344-31546113	14	9	9	64
VEGFA	6	43737946-43754224	49	6	24	49
IL6	7	22766766-22771621	17	3	9	53
SERPINE1	7	100770370-100782547	39	6	20	51
NAMPT	7	105888731-105925638	51	8	44	86
ANGPT2	8	6357172-6420784	357	56	291	82
MBL2	10	54525140-54531460	42	9	27	64
PLAU	10	75670862-75677259	15	2	5	33
SFTPA2	10	81315608-81320163	48	0	0	0
SFTPA1	10	81370695-81375199	49	0	0	0

SFTPD	10	81697496-81708861	44	6	20	45
FAS	10	90748288-90777542	101	17	99	98
TRAF6	11	36505317-36531863	41	7	23	56
IL18	11	112013974-112034840	38	5	28	74
TIRAP	11	126152982-126164828	38	5	15	39
IRAK3	12	66582978-66648402	83	11	55	66
NFKBIA	14	35870716-35873960	28	2	2	7
DIO2	14	80663868-80697397	46	7	39	85
IL32	16	3115313-3119668	41	4	5	12
HMOX2	16	4524719-4560348	50	5	43	86
NQO1	16	69743304-69760533	39	6	21	54
ACE	17	61554422-61575741	48	7	36	75
FTL	19	49468566-49470136	4	1	2	50
PI3	20	43803540-43805185	26	1	24	92
MIF	22	24236565-24237409	24	3	19	79
HMOX1	22	35777060-35790207	62	3	38	61

[a]According to NCBI build 37.
[b]Number of SNPs from the gene region with minor allele frequency >5% reported for Europeans from the 1000 Genomes Project (data as May 2011).
[c]Number of SNPs with minor allele frequency >5% in the gene region captured by the array both directly and indirectly ($r^2 \geq 0.8$). The tagger tool (Barrett, 2005) was used to perform this calculation using aggressive tagging.
[d]SNPs with minor allele frequency >5% covered by the study considering indirectly tested variants ([SNPs indirectly tested÷Common SNPs]*100).

2. References

Arcaroli, J.J., Hokanson, J.E., Abraham, E., Geraci, M., Murphy, J.R., Bowler, R.P., Dinarello, C.A., Silveira, L., Sankoff, J., Heyland, D., Wischmeyer, P., and Crapo, J.D. (2009). Extracellular superoxide dismutase haplotypes are associated with acute lung injury and mortality. *Am J Respir Crit Care Med* 179**,** 105-112.

Arcaroli, J.J., Liu, N., Yi, N., and Abraham, E. (2011). Association between IL-32 genotypes and outcome in infection-associated acute lung injury. *Crit Care* 15**,** R138.

Bajwa, E.K., Cremer, P.C., Gong, M.N., Zhai, R., Su, L., Thompson, B.T., and Christiani, D.C. (2011). An NFKB1 promoter insertion/deletion polymorphism influences risk and outcome in acute respiratory distress syndrome among Caucasians. *PLoS One* 6**,** e19469.

Barrett, J.C., Fry, B., Maller, J., and Daly, M.J. (2005). Haploview: analysis and visualization of LD and haplotype maps. *Bioinformatics* 21, 263-265.

Chen, S., Xu, L., and Tang, J. (2010). Association of interleukin 18 gene polymorphism with susceptibility to the development of acute lung injury after cardiopulmonary bypass surgery. *Tissue Antigens* 76**,** 245-249.

Garcia-Laorden, M.I., Rodriguez De Castro, F., Sole-Violan, J., Rajas, O., Blanquer, J., Borderias, L., Aspa, J., Briones, M.L., Saavedra, P., Marcos-Ramos, J.A., Gonzalez-Quevedo, N., Sologuren, I., Herrera-Ramos, E., Ferrer, J.M., Rello, J., and Rodriguez-Gallego, C. (2011). Influence of genetic variability at the surfactant proteins A and D in community-acquired pneumonia: a prospective, observational, genetic study. *Crit Care* 15**,** R57.

Glavan, B.J., Holden, T.D., Goss, C.H., Black, R.A., Neff, M.J., Nathens, A.B., Martin, T.R., and Wurfel, M.M. (2010). Genetic variation in the FAS gene and associations with acute lung injury. *Am J Respir Crit Care Med* 183**,** 356-363.

Jin, X., Hu, Z., Kang, Y., Liu, C., Zhou, Y., Wu, X., Liu, J., Zhong, M., Luo, C., Deng, L., Deng, Y., Xie, X., Zhang, Z., Zhou, Y., and Liao, X. (2011). Association of interleukin-10-1082 G/G genotype with lower mortality of acute respiratory distress syndrome in a Chinese population. *Genet Test Mol Biomarkers* 15**,** 203-206.

Kangelaris, K.N., Sapru, A., Calfee, C.S., Liu, K.D., Pawlikowska, L., Witte, J.S., Vittinghoff, E., Zhuo, H., Auerbach, A.D., Ziv, E., and Matthay, M.A. (2012). The association between a Darc gene polymorphism and clinical outcomes in African American patients with acute lung injury. *Chest* 141**,** 1160-1169.

Lagan, A.L., Quinlan, G.J., Mumby, S., Melley, D.D., Goldstraw, P., Bellingan, G.J., Hill, M.R., Briggs, D., Pantelidis, P., Du Bois, R.M., Welsh, K.I., and Evans, T.W. (2008). Variation in iron homeostasis genes between patients with ARDS and healthy control subjects. *Chest* 133**,** 1302-1311.

Liu, L.X., You, X.J., Zhang, Y.X., Zhao, C., Chen, L., and Hu, Z.J. (2009). [Relationship between tumor necrosis factor beta gene polymorphism and acute respiratory distress syndrome after operation for esophageal carcinoma]. *Ai Zheng* 28**,** 1255-1259.

Lu, X.M., Chen, G.J., Yang, Y., and Qiu, H.B. (2011). Angiotensin-converting enzyme

polymorphism affects outcome of local Chinese with acute lung injury. *Respir Med* 105**,** 1485-1490.

Ma, S.F., Xie, L., Pino-Yanes, M., Sammani, S., Wade, M.S., Letsiou, E., Siegler, J., Wang, T., Infusino, G., Kittles, R.A., Flores, C., Zhou, T., Prabhakar, B.S., Moreno-Vinasco, L., Villar, J., Jacobson, J.R., Dudek, S.M., and Garcia, J.G. (2011). Type 2 deiodinase and host responses of sepsis and acute lung injury. *Am J Respir Cell Mol Biol* 45**,** 1203-1211.

Martin-Loeches, I., Sole-Violan, J., Rodriguez De Castro, F., Garcia-Laorden, M.I., Borderias, L., Blanquer, J., Rajas, O., Briones, M.L., Aspa, J., Herrera-Ramos, E., Marcos-Ramos, J.A., Sologuren, I., Gonzalez-Quevedo, N., Ferrer-Aguero, J.M., Noda, J., and Rodriguez-Gallego, C. (2011). Variants at the promoter of the interleukin-6 gene are associated with severity and outcome of pneumococcal community-acquired pneumonia. *Intensive Care Med* 38**,** 256-262.

Meyer, N.J., Daye, Z.J., Rushefski, M., Aplenc, R., Lanken, P.N., Shashaty, M.G., Christie, J.D., and Feng, R. (2012). SNP-set analysis replicates acute lung injury genetic risk factors. *BMC Med Genet* 13**,** 52.

Meyer, N.J., Li, M., Feng, R., Bradfield, J., Gallop, R., Bellamy, S., Fuchs, B.D., Lanken, P.N., Albelda, S.M., Rushefski, M., Aplenc, R., Abramova, H., Atochina-Vasserman, E.N., Beers, M.F., Calfee, C.S., Cohen, M.J., Pittet, J.F., Christiani, D.C., O'keefe, G.E., Ware, L.B., May, A.K., Wurfel, M.M., Hakonarson, H., and Christie, J.D. (2011). ANGPT2 genetic variant is associated with trauma-associated acute lung injury and altered plasma angiopoietin-2 isoform ratio. *Am J Respir Crit Care Med* 183**,** 1344-1353.

Pino-Yanes, M., Corrales, A., Casula, M., Blanco, J., Muriel, A., Espinosa, E., Garcia-Bello, M., Torres, A., Ferrer, M., Zavala, E., Villar, J., and Flores, C. (2010). Common variants of TLR1 associate with organ dysfunction and sustained pro-inflammatory responses during sepsis. *PLoS One* 5**,** e13759.

Pino-Yanes, M., Ma, S.F., Sun, X., Tejera, P., Corrales, A., Blanco, J., Perez-Mendez, L., Espinosa, E., Muriel, A., Blanch, L., Garcia, J.G., Villar, J., and Flores, C. (2011). Interleukin-1 receptor-associated kinase 3 gene associates with susceptibility to acute lung injury. *Am J Respir Cell Mol Biol* 45**,** 740-745.

Reddy, A.J., Christie, J.D., Aplenc, R., Fuchs, B., Lanken, P.N., and Kleeberger, S.R. (2009). Association of human NAD(P)H:quinone oxidoreductase 1 (NQO1) polymorphism with development of acute lung injury. *J Cell Mol Med* 13**,** 1784-1791.

Sheu, C.C., Zhai, R., Su, L., Tejera, P., Gong, M.N., Thompson, B.T., Chen, F., and Christiani, D.C. (2009a). Sex-specific association of epidermal growth factor gene polymorphisms with acute respiratory distress syndrome. *Eur Respir J* 33**,** 543-550.

Sheu, C.C., Zhai, R., Wang, Z., Gong, M.N., Tejera, P., Chen, F., Su, L., Thompson, B.T., and Christiani, D.C. (2009b). Heme oxygenase-1 microsatellite polymorphism and haplotypes are associated with the development of acute respiratory distress syndrome. *Intensive Care Med* 35**,** 1343-1351.

Song, Z., Tong, C., Sun, Z., Shen, Y., Yao, C., Jiang, J., Yin, J., Gao, L., Song, Y., and Bai, C. (2010). Genetic variants in the TIRAP gene are associated with increased risk of sepsis-associated acute lung injury. *BMC Med Genet* 11**,** 168.

Song, Z., Yao, C., Yin, J., Tong, C., Zhu, D., Sun, Z., Jiang, J., Shao, M., Zhang, Y., Deng, Z., Tao, Z., Sun, S., and Bai, C. (2012). Genetic variation in the TNF receptor-associated factor 6 gene is associated with susceptibility to sepsis-induced acute lung injury. *J Transl Med* 10**,** 166.

Su, L., Zhai, R., Sheu, C.C., Gallagher, D.C., Gong, M.N., Tejera, P., Thompson, B.T., and Christiani, D.C. (2009). Genetic variants in the angiopoietin-2 gene are associated with increased risk of ARDS. *Intensive Care Med* 35**,** 1024-1030.

Tejera, P., Wang, Z., Zhai, R., Su, L., Sheu, C.C., Taylor, D.M., Chen, F., Gong, M.N., Thompson, B.T., and Christiani, D.C. (2009). Genetic polymorphisms of peptidase inhibitor 3 (elafin) are associated with acute respiratory distress syndrome. *Am J Respir Cell Mol Biol* 41**,** 696-704.

Tsangaris, I., Tsantes, A., Bonovas, S., Lignos, M., Kopterides, P., Gialeraki, A., Rapti, E., Orfanos, S., Dimopoulou, I., Travlou, A., and Armaganidis, A. (2009). The impact of the PAI-1 4G/5G polymorphism on the outcome of patients with ALI/ARDS. *Thromb Res* 123**,** 832-836.

Wurfel, M.M., Gordon, A.C., Holden, T.D., Radella, F., Strout, J., Kajikawa, O., Ruzinski, J.T., Rona, G., Black, R.A., Stratton, S., Jarvik, G.P., Hajjar, A.M., Nickerson, D.A., Rieder, M., Sevransky, J., Maloney, J.P., Moss, M., Martin, G., Shanholtz, C., Garcia, J.G., Gao, L., Brower, R., Barnes, K.C., Walley, K.R., Russell, J.A., and Martin, T.R. (2008). Toll-like receptor 1 polymorphisms affect innate immune responses and outcomes in sepsis. *Am J Respir Crit Care Med* 178**,** 710-720.

Yang, S., Cao, S., Li, J., and Chang, J. (2011). Association between vascular endothelial growth factor + 936 genotype and acute respiratory distress syndrome in a Chinese population. *Genet Test Mol Biomarkers* 15**,** 737-740.
